# Hematologic changes after short term hypoxia in non-elite apnea divers under voluntary dry apnea conditions

**DOI:** 10.1371/journal.pone.0237673

**Published:** 2020-08-13

**Authors:** Ramona C. Dolscheid-Pommerich, Birgit Stoffel-Wagner, Judith Alberts, Rolf Fimmers, Lars Eichhorn

**Affiliations:** 1 Institute of Clinical Chemistry and Clinical Pharmacology, University Hospital Bonn, Bonn, Germany; 2 Department of Anesthesiology and Intensive Care Medicine, University Hospital Bonn, Bonn, Germany; 3 Institute for Medical Biometry, Informatics and Epidemiology, University Hospital Bonn, Bonn, Germany; The Ohio State University, UNITED STATES

## Abstract

**Purpose:**

This study investigated the acute changes in full spectrum differential blood cell count including reticulocytes and immature reticulocytes after a voluntary maximal dry apnea in non-elite divers. Aim of the present study is to obtain information on important regulatory compensation mechanisms and to provide insights into apneic regulatory processes.

**Methods:**

Ten apnea divers performed a voluntary dry mean apnea time of 317 sec [SD ±111 sec]. Differential blood cell count including reticulocytes was measured before and immediately after a single maximal breath-hold. To evaluate kinetics, blood samples were also taken after 30 min and 4 h. Value distributions are presented with dot plots. *P*-values were calculated using a mixed linear model for time dependency. Four difference values were compared to baseline values with Dunnett’s procedure.

**Results:**

Significant changes were found in red blood cell parameters for erythrocytes, red cell distribution width, hematocrit, hemoglobin, MCV, reticulocytes and immature reticulocytes, and in white blood cell parameters for leucocytes, lymphocytes, immature granulocytes, monocytes, basophile granulocytes, neutrophil granulocytes and eosinophil granulocytes and for thrombocytes.

**Conclusion:**

Adaptive mechanisms regarding cell counts in elite apnea divers are not readily transferable to non-elite recreational sportspersons. Divers and physicians should be aware of the limited adaptive performance of humans in the case of extended apnea.

## Introduction

Breath-hold or apnea diving’s main characteristic is a state of hypercapnic hypoxia. Early studies introduced the diving response with a signature combination of peripheral vasoconstriction based on sympathetic nervous system activity in combination with bradycardia and lower cardiac output [1,2]. Apnea diving triggers several different reactions: Hypoxia during the apneic phase leads to modifications in the hormonal regulation of erythropoiesis. In elite breath-hold divers, significant increases in erythropoietin levels were reported at 0.5 h and 2.5 h compared to baseline values [3]. Post apnea hemoglobin levels were increased in elite apneic divers [4]. Markers for endothelial dysfunction were also increased after a single breath-hold phase [5]. Oxidative stress is increased in breath-hold divers. Theunissen et al. reported that after the apneic phase, an elevation in the stress level markers, plasma nitric oxide and peroxinitrites as well as a reduction in thiols occurred [6]. Different markers of stress and injury also showed elevated levels: Marlinge et al. observed increased levels of copeptin and ischemia modified albumin in dry static apnea [7]. Not only elevated values of catecholamines, but also elevated levels of troponin and brain natriuretic peptide were detected [8].

While apnea results in multiple physiological and pathophysiological changes, it seems relevant to determine whether maximal apnea in trained divers has an impact on the three blood cell lines. For leucocytes, this question arises since leucocytes play an important role in our immune system, and also because stress has a great impact on leucocyte distribution [9]. To examine the full spectrum of red blood cell distribution, not only hemoglobin and hematocrit should be examined [3,7], but also other parameters, such as red blood cells and precursor immature reticulocytes and reticulocytes. Redistribution mechanisms and hemoconcentration take place during the apneic phase [10]. Activation of thrombocytes as primary hemostasis might be detectable due to micro-bleedings or hemoptysis [11,12]. Accordingly, this study examines the full spectrum of differential blood cell count including precursor cells, such as immature granulocytes, reticulocytes and immature reticulocytes. The investigation of the degree of integration of the three blood cell lines may reveal information about important regulatory compensation mechanisms which possibly occur after breath-hold diving. We propose that cell count as well as differential blood cell count parameters are influenced by single short-term hypoxia under dry apnea conditions. Aim of the study is to provide further insights into apneic regulatory or pathophysiological processes.

## Materials and methods

### Single-center prospective study

In accordance with the 1964 Helsinki declaration and its later amendments, this study received approval from the local ethics committee (Ethikkommission an der Medizinischen Fakultät der Rheinischen Friedrich-Wilhelms-Universität Bonn, Chairman K. Racké, MD, PhD, Professor, University Clinics Bonn). Participants were originally recruited in 2014 for a study to evaluate the influence of apnea on cardiac function using MRI. Inclusion criteria were minimum age of 18 years, breath-hold time of >270 s, absence of long-term medication or history of any cardiac and lung diseases. All study participants received an information sheet 14 days prior to the study and written consent was obtained. After one year, participants were re-examined for follow-up studies. Participants were required to abstain from caffeine containing drinks and food at least 8 hrs prior to the examination. During this follow-up in 2015, participants performed a maximal dry apnea in a horizontal position under dry conditions. Blood was immediately transported to the central laboratory of the University Clinics Bonn. In all samples, analysis was performed within 15 min of arrival at the central laboratory.

### Materials and data

The mean age of the participants (eight male, two female) was 41 years (± 10; range 25–54). Mean bodyweight was 84 kg (± 12; range 68–110), and mean height was 183 cm (± 9; range 166–200) resulting in a mean Body Mass Index of 24.94 (range 21.95–28.34). Mean apnea time was 329 seconds (± 103; range 168–587). Heart frequency dropped from 86 bmp (± 12; range 70–115) to 51 bmp (±11; range 32–77). SpO2 dropped from 99% (± 0.5; range 99–100) before apnea to 79% (± 12, range 56–96) at the end of apnea.

Venous EDTA whole blood samples were taken before apnea, immediately after apnea, and 0.5 h and 4 h after maximal dry apnea. Blood was immediately transported to the central laboratory of the University Clinics Bonn. In all samples, analysis was performed within 15 min of arrival at the central laboratory. Levels of erythrocytes, red cell distribution width (RDW), leucocytes, thrombocytes, lymphocytes, basophile granulocytes, neutrophil granulocytes, eosinophil granulocytes, monocytes, immature granulocytes, reticulocytes, immature reticulocytes, reticulocyte hemoglobin, reticulocyte production index, hemoglobin, hematocrit, mean corpuscular volume, mean corpuscular hemoglobin, mean corpuscular hemoglobin concentration and mean platelet volume were determined (cells as absolute and percental count). S1 File showing raw data. Values were measured with Sysmex XN1000^™^ (Sysmex, Norderstedt, Germany). Hemoglobin (Hb) concentration was photometrically measured with the sodium lauryl sulfate hemoglobin method, erythrocytes and thrombocytes were measured with impedance technique. Hematocrit (Hk) and mean corpuscular volume (MCV) were measured with summarizing impulse heights, while mean corpuscular hemoglobin (MCH) and mean corpuscular hemoglobin concentration (MCHC) were computed. In the Sysmex WDF channel, lymphocytes, monocytes, neutrophil granulocytes and eosinophil granulocytes were measured with fluorescence flow cytometry. In the Sysmex WNR channel, basophil granulocytes were measured. Reticulocytes and immature reticulocytes were determined with fluorescence flow cytometry in a specific RET channel. Methods for differential blood cell count, reticulocytes and immature reticulocytes at the central laboratory are accredited according to DIN EN ISO 15189:2014. Analyses were performed in line with the guideline of the German Medical Association (RiliBÄK) according to stipulated internal and external quality controls.

### Statistics

For statistical analysis, SAS^®^ (SAS Institute Inc., Cary, NC, USA. Version 9.4.) was used. Parameters were described with mean values ± SD. Dot plots show the value distributions of the analyzed parameters for the four different time points. To analyze time dependency of differential blood cell count parameters, reticulocytes and immature reticulocytes, values were analyzed using a mixed linear model taking the proband as random effect. According to Dunnett’s procedure (adjusted for multiple testing per variable), baseline differences with adjusted confidence limits at the three follow-up time points were calculated.

## Results

### Red blood cells

**Fig 1:**
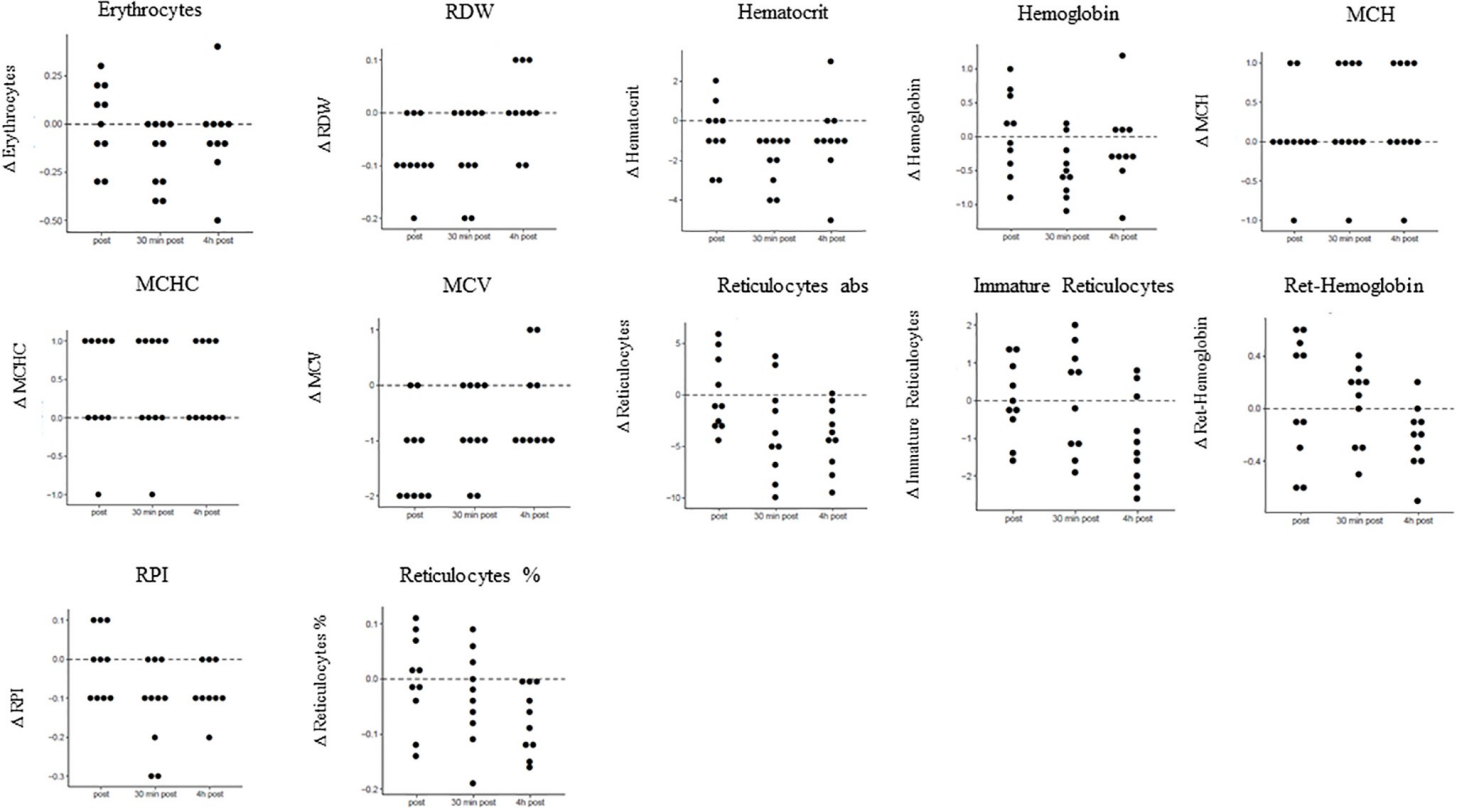
Concentration changes of red blood cell parameters compared to baseline. Displayed are concentration changes at the three different time points post apnea, 30 min and 4 h post apnea compared to baseline values for red blood cell parameters.

Mean values and SD of erythrocytes, red cell distribution width, reticulocytes, immature reticulocytes, reticulocyte hemoglobin, reticulocyte production index, hemoglobin, hematocrit, mean corpuscular volume, mean corpuscular hemoglobin and mean corpuscular hemoglobin concentration were calculated before apnea, and immediately and 0.5 h and 4 h after apnea. Significant time dependencies were found for erythrocytes, red cell distribution width, Hk, Hb, MCV, reticulocytes and immature reticulocytes. *P*-values as well as mean values ± SD for baseline concentration, before apnea, and immediately and 0.5 h and 4 h after apnea are presented in Table 1. Differences between the three time intervals versus baseline concentrations for red blood cell parameters are displayed in Table 2. Fig 1 dot plots showing the value distributions of red blood cell parameters for the different time points (Fig 1).

**Table 1:** Mean values, standard deviations and *p*-values at four different time points (baseline concentrations, immediately post apnea, 0.5 h and 4 h after apnea) of red blood cell parameters.

Mean [± SD]	*p*-value	Baseline concentration [± SD]	Post apnea [± SD]	0.5 h post apnea [± SD]	4 h post apnea [± SD]
Erythrocytes T/l	**0.0143**	4.7 [±0.4]	4.7 [±0.4]	4.6 [±0.4]	4.7 [±0.5]
RDW %	**0.0033**	12.3 [±0.6]	12.2 [±0.6]	12.2 [±0.6]	12.3 [±0.6]
Hematocrit %	**0.0014**	40.9 [±2.6]	40.3 [±3.2]	38.9 [±2.8]	40.0 [±3.3]
Hemoglobin g/dl	**0.0065**	14.3 [±1.1]	14.4 [±1.3]	13.8 [±1.1]	14.2 [±1.4]
MCH pg	0.2394	30.3 [±1.9]	30.4 [±2.1]	30.6 [±1.8]	30.6 [±1.9]
MCHC g/dl	0.1322	35.1 [±1.1]	35.5 [±1.0]	35.5 [±0.8]	35.5 [±1.1]
MCV fl	**<0.0001**	86.5 [±3.9]	85.2 [±3.7]	85.7 [±3.6]	86.1 [±4.0]
Reticulocytes absolute G/l	**0.0029**	57.4 [±21.3]	57.4 [±19.7]	54.0 [±18.3]	53.3 [±20.4]
Reticulocytes %	**0.0109**	1.2 [±0.5]	1.2 [±0.5]	1.2 [±0.4]	1.2 [±0.5]
Ret-Hemoglobin pg	0.0624	32.6 [±1.8]	32.6 [±1.9]	32.6 [±1.7]	32.3 [±1.8]
Immature Reticulocytes %	**0.0163**	5.6 [±1.7]	5.6 [±1.8]	5.6 [±2.2]	4.5 [±2.1]
RPI	**0.0012**	1.1 [±0.4]	1.1 [±0.4]	1.0 [±0.4]	1.0 [±0.4]

Mean values, standard deviations (SD) and *p*-values at the four time points baseline concentrations, immediately post apnea, 0.5 h and 4 h after apnea for erythrocytes, RDW, hematocrit, hemoglobin, MCH, MCHC, MCV, reticulocytes, ret-hemoglobin, immature reticulocytes and RPI. Significant *p*-values are marked in bold.

**Table 2:** Differences between three time intervals versus baseline concentrations for red blood cell parameters.

Red blood cell parameter	*p*-value post apnea vs. baseline concentration (mean value [CI])	*p*-value 0.5 h post apnea vs. baseline concentration (mean value [CI])	*p*-value 4 h post apnea vs. baseline concentration (mean value [CI])
Erythrocytes T/l	0.9954 (0.01 [-0.1002;0.1202])	**0.0164** (-0.16 [-0.2702;-0.04975])	0.5519 (-0.06 [-0.1702;0.05025])
RDW %	**0.0179** (0.02722 [-0.1358;-0.02416])	**0.0416** (0.02722 [-0.1258;-0.01416])	0.9672 (0.02722 [-0.04584;0.06584])
Hematocrit %	0.4201 (-0.6 [-1.5290;0.329])	**0.0004** (-2.0 [-2.9290;-1.071])	0.1391 (-0.9 [-1.8290;0.02901])
Hemoglobin g/dl	0.9751 (0.05 [-0.2581;0.3581]	**0.0097** (-0.48 [-0.7881;-0.1719])	0.6766 (-0.14 [-0.4481;0.1681])
MCH pg	0.8913 (0.1 [-0.2565;0.4565])	0.2234 (0.3 [-0.05648;0.6565])	0.2234 (0.3 [-0.05648;0.6565])
MCHC g/dl	0.1311 (0.4 [-0.00655;0.8065])	0.1311 (0.4 [-0.00655;0.8065])	0.1311 (0.4 [-0.00655;0.8065])
MCV fl	**<0.0001** (-1.3 [-1.7828;-0.8172])	**0.0058** (-0.8 [-1.2828;-0.3172])	0.2340 (-0.4 [-0.8828;0.08281])
Reticulocytes G/l	1.0000 (-888e^-18^ [-2.6167;2.6167])	**0.0298** (-3.47 [-6.0867;-0.8533])	**0.0089** (-4.12 [-6.7367;-1.5033])
Reticulocytes %	0.9984 (-0.003 [-0.05057;0.04457])	0.3883 (-0.032 [-0.07957;0.01557])	**0.0088** (-0.075 [-0.1226;-0.02743])
Ret-Hemoglobin pg	0.8208 (0.08 [-0.152;0.312])	0.9870 (0.03 [-0.202;0.262])	0.1506 (-0.22 [-0.452;0.01199])
Immature Reticulocytes %	1.0000 (9.537e^-8^ [-0.7445;0.7445])	0.9999 (0.02 [-0.7245;0.7645])	**0.0227** (-1.03 [-1.7745;-0.2855])
RPI	0.9775 (-0.01 [-0.07365;0.05365])	**0.0018** (-0.12 [-0.1837;-0.05635])	**0.0285** (-0.08822 [-0.1542;-0.02226])

*p*-values, mean values and confidence intervals (CI) for change for the differences between the three time intervals post apnea, 0.5 h and 4 h post apnea versus baseline concentrations for erythrocytes, RDW, hematocrit, hemoglobin, MCH, MCHC, MCV, reticulocytes, ret-hemoglobin, immature reticulocytes and RPI. Significant *p*-values are marked in bold.

### White blood cells

**Fig 2:**
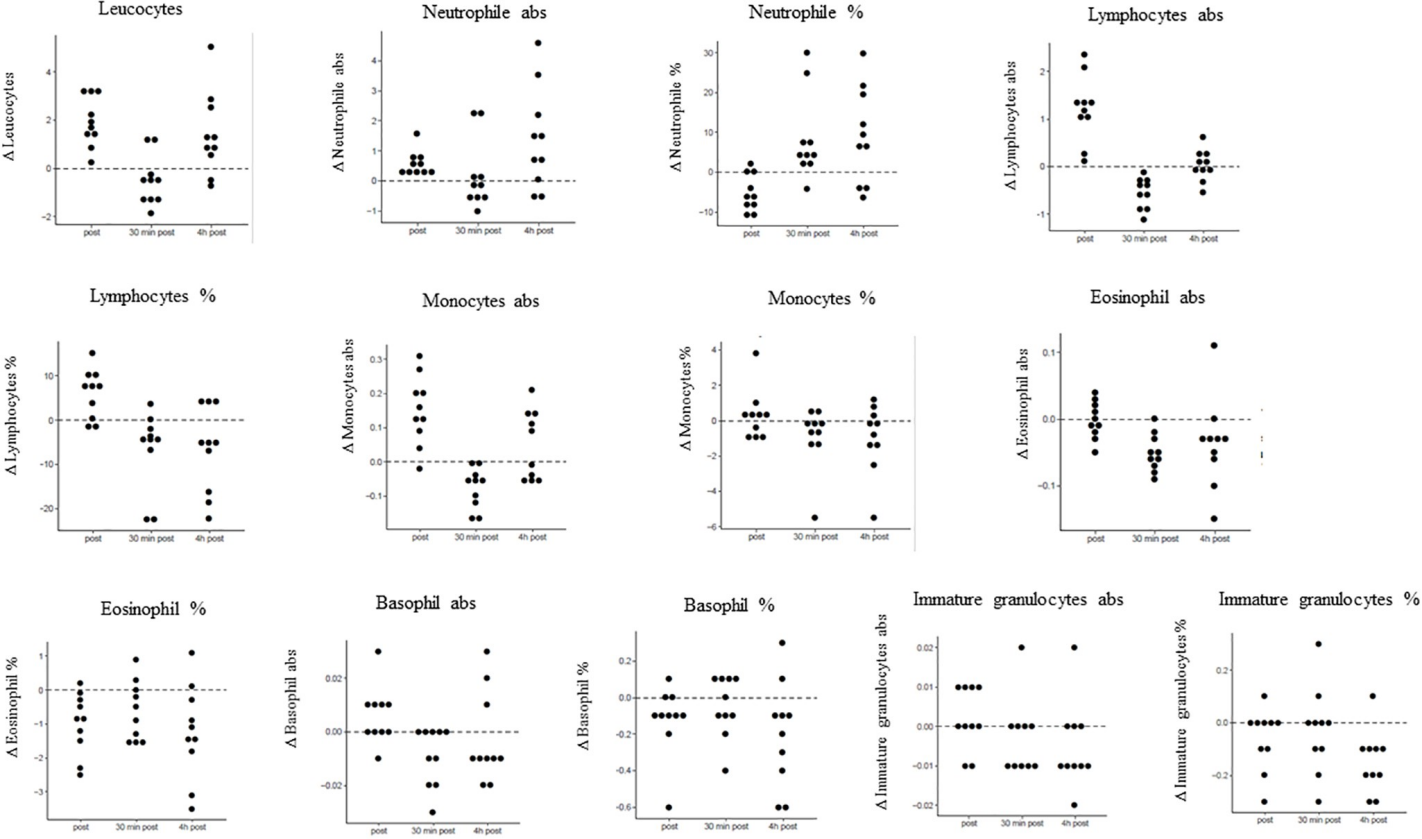
Concentration changes of white blood cell parameters compared to baseline. Displayed are concentration changes at the three different time points post apnea, 30 min and 4 h post apnea compared to baseline values for white blood cell parameters.

Mean values and SD of leucocytes, lymphocytes, basophile granulocytes, neutrophil granulocytes, eosinophil granulocytes, immature granulocytes and monocytes were calculated before apnea, and immediately, 0.5 h and 4 h after apnea. Significant time dependencies were found for leucocytes, lymphocytes, immature granulocytes, monocytes, basophile granulocytes, neutrophil granulocytes and eosinophil granulocytes. *P*-values as well as mean values ± SD for baseline concentration, before apnea, and immediately and 0.5 h and 4 h after apnea are presented in Table 3. Differences between the three time intervals versus baseline concentrations for white blood cell parameters are displayed in Table 4. Fig 2 presents dot plots showing the value distributions of white blood cell parameters for the different time points (Fig 2).

**Table 3:** Mean values, standard deviations and *p*-values at four different time points (baseline concentrations, immediately post apnea, 0.5 h and 4 h after apnea) of white blood cell parameters.

Mean [± SD]	*p*-value	Baseline concentration [± SD]	Post apnea [± SD]	0.5 h post apnea [± SD]	4 h post apnea [± SD]
Leucocytes G/l	**<0.0001**	5.5 [±1.3]	7.4 [±1.7]	5.0 [±1.1]	6.9 [±1.7]
Neutrophil granulocytes G/l	**0.0045**	2.8 [±0.9]	3.4 [±0.9]	3.0 [±1.0]	4.2 [±1.5]
Neutrophil granulocytes %	**<0.0001**	50.2 [±7.2]	45.0 [±5.1]	58.5 [±9.1]	59.3 [±10.6]
Lymphocytes G/l	**<0.0001**	2.1 [±0.7]	3.3 [±1.0]	1.6 [±0.5]	2.1 [±0.8]
Lymphocytes %	**<0.0001**	38.2 [±6.0]	44.1 [±5.0]	31.5 [±8.7]	31.4 [±10.9]
Monocytes G/l	**<0.0001**	0.4 [±0.1]	0.5 [±0.1]	0.3 [±0.1]	0.4 [±0.1]
Monocytes %	0.0560	7.6 [±2.6]	7.9 [±3.0]	6.7 [±1.9]	6.6 [±1.9]
Eosinophil granulocytes G/l	**0.0014**	0.2 [±0.1]	0.2 [±0.1]	0.1 [±0.1]	0.1 [±0.1]
Eosinophil granulocytes %	**0.0053**	3.3 [±2.1]	2.3 [±1.3]	2.6 [±2.3]	2.0 [±1.5]
Basophil granulocytes G/l	**0.0248**	0.05 [±0.03]	0.05 [±0.04]	0.04 [±0.02]	0.04 [±0.03]
Basophil granulocytes %	0.0595	0.8 [±0.5]	0.7 [±0.4]	0.8 [±0.5]	0.6 [±0.4]
Immature granulocytes G/l	0.0841	0.02 [±0.007]	0.02 [±0.007]	0.01 [±0.01]	0.01 [±0.008]
Immature granulocytes %	**0.0178**	0.3 [±0.1]	0.3 [±0.1]	0.3 [±0.2]	0.2 [±0.1]

Mean values, standard deviations (SD) and *p*-values at the four time points baseline concentrations, immediately post apnea, 0.5 h and 4 h after apnea for leucocytes, neutrophil granulocytes, lymphocytes, monocytes, eosinophil granulocytes, basophil granulocytes and immature granulocytes. Significant *p*-values are marked in bold.

**Table 4:** Differences between three time intervals versus baseline concentrations for white blood cell parameters.

White blood cell parameter	*p*-value post apnea vs. baseline concentration (mean value [CI])	*p*-value 0.5 h post apnea vs. baseline concentration (mean value [CI])	*p*-value 4 h post apnea vs. baseline concentration (mean value [CI])
Leucocytes G/l	**0.0003** (1.934 [1.0546;2.8134])	0.4919 (-0.518 [-1.3974;0.3614])	**0.0079** (1.404 [0.5246;2.2834])
Neutrophil granulocytes G/l	0.2962 (0.573 [-0.182;1.328])	0.9337 (0.175 [-0.58;0.93])	**0.0026** (1.369 [0.614;2.124])
Neutrophil granulocytes %	0.1806 (-5.15 [-10.8707;0.5707])	**0.0167** (8.28 [2.5593;14.0007])	**0.0079** (9.14 [3.4193;14.8607])
Lymphocytes G/l	**<0.0001** (1.207 [0.869;1.545])	**0.0061** (-0.557 [-0.895;-0.219])	0.9968 (0.027 [-0.311;0.365])
Lymphocytes %	**0.0461** (5.96 [1.1183;10.8017])	**0.0224** (-6.71 [-11.5517;-1.8683])	**0.0218** (-6.74 [-11.5817;-1.8983])
Monocytes G/l	**<0.0001** (0.15 [0.09702;0.203])	**0.0178** (-0.076 [-0.129;-0.02302])	0.1768 (0.048 [-0.00498;0.101])
Monocytes %	0.8949 (0.3 [-0.7839;1.3839])	0.2479 (-0.88 [-1.9639;0.2039])	0.1905 (-0.96 [-2.0439;0.1239])
Eosinophil granulocytes G/l	0.9978 (-0.002 [-0.03018;0.02618])	**0.0026** (-0.051 [-0.07918;-0.02282])	**0.0316** (-0.037 [-0.06518;-0.00882])
Eosinophil granulocytes %	**0.0157** (-0.99 [-1.6681;-0.3119])	0.1532 (-0.64 [-1.3181;0.03815])	**0.0024** (-1.24 [-1.9181;-0.5619])
Basophil granulocytes G/l	0.4357 (0.006 [-0.00348; 0.01548])	0.1498 (-0.009 [-0.01848;0.000477])	0.8539 (-0.003 [-0.01248;0.006477])
Basophil granulocytes %	0.2627 (-0.12 [-0.2710;0.03096])	0.8369 (-0.05 [-0.201;0.101])	**0.0299** (-0.2 [-0.351;-0.04904])
Immature granulocytes G/l	0.8172 (0.002 [-0.00375;0.007749])	0.5827 (-0.003 [-0.00875;0.002749])	0.2020 (-0.005 [-0.01075;0.000749])
Immature granulocytes %	0.4303 (-0.06 [-0.1541;0.03411])	0.8511 (-0.03 [-0.1241;0.06411])	**0.0081** (-0.15 (-0.2441;-0.05589])

*p*-values, mean values and confidence intervals (CI) for change for the differences between the three time intervals post apnea, 0.5 h and 4 h post apnea versus baseline concentrations for leucocytes, neutrophil granulocytes, lymphocytes, monocytes, eosinophil granulocytes, basophil granulocytes and immature granulocytes. Significant *p*-values are marked in bold.

### Thrombocytes

**Fig 3:**
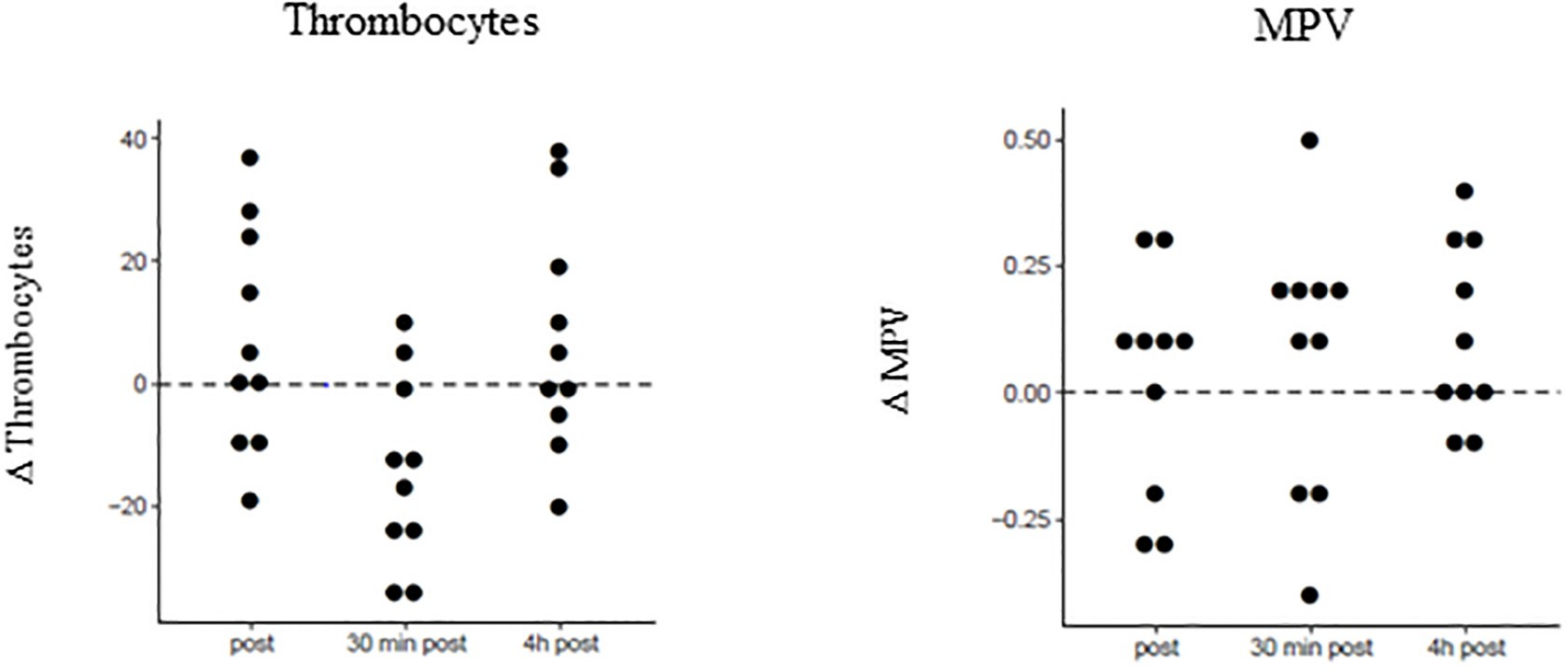
Concentration changes of thrombocytes and mean platelet volume compared to baseline. Displayed are concentration changes at different time points post apnea, 30 min and 4 h post apnea compared to baseline values for thrombocytes and mean platelet volume.

Mean values and SD of thrombocytes and mean platelet volume were calculated before apnea, and immediately and 0.5 h and 4 h after apnea. Significant time dependencies were found for thrombocytes. *P*-values as well as mean values ± SD for baseline concentration, before apnea, and immediately and 0.5 h and 4 h after apnea are presented in Table 5. Differences between the three time intervals versus baseline concentrations for thrombocytes and mean platelet volume are displayed in Table 6. Fig 3 presents dot plots showing the value distributions of thrombocytes and mean platelet volume for the different time points (Fig 3).

**Table 5:** Mean values, standard deviations and *p*-values at four different time points (baseline concentrations, immediately post apnea, 0.5 h and 4 h after apnea) for thrombocytes and mean platelet volume.

Mean [± SD]	*p*-value	Baseline concentration [± SD]	Post apnea [± SD]	0.5 h post apnea [± SD]	4 h post apnea [± SD]
Thrombocytes G/l	**0.0003**	221.4 [±38.0]	228.5 [±46.5]	207.0 [±42.1]	228.4 [±49.6]
MPV	0.4046	10.4 [±0.9]	10.5 [±0.9]	10.5 [±0.9]	10.5 [±0.8]

Mean values, standard deviations (SD) and *p*-values at the four time points baseline concentrations, immediately post apnea, 0.5 h and 4 h after apnea for thrombocytes and mean platelet volume. Significant *p*-values are marked in bold.

**Table 6:** Differences between three time intervals versus baseline concentrations for thrombocytes and mean platelet volume.

Thrombocyte parameter	*p*-value Post apnea vs. baseline concentration (mean value [CI])	*p*-value 0.5 h post apnea vs. baseline concentration (mean value [CI])	*p*-value 4 h post apnea vs. baseline concentration (mean value [CI])
Thrombocytes G/l	0.3378 (7.1 [-2.7873;16.9873])	**0.0160** (-14.4 [-24.2873;-4.5127])	0.3487 (7.0 [-2.8873;16.8873])
MPV	0.9839 (0.02 [-0.1236;0.1636])	0.6303 (0.07 [-0.07357;0.2136])	0.2893 (0.11 [-0.03357;0.2536])

*p*-values, mean values and confidence intervals (CI) for change for the differences between the three time intervals post apnea, 0.5 h and 4 h post apnea versus baseline concentrations of thrombocytes and mean platelet volume. Significant *p*-values are marked in bold.

## Discussion

This study examines the full spectrum differential blood cell count including precursor cells immature granulocytes, reticulocytes and reticulocyte parameters after voluntary maximal dry apnea. The investigation showed that even a single maximal apnea leads to significant changes in all three blood cell lines.

Spleen contraction is known to autotransfuse a large quantity of red blood cells into the systemic circulation, thereby augmenting the blood’s oxygen transport capacity. This augmentation allows diving mammals to dive for a longer period of time [13], it possibly improves performance in running mammals like horses or dogs [14,15], and it can also be seen in humans in case of diving [16]. It has been shown that eupneic normobaric hypoxia as well as increased CO_2_ in the absence of hypoxia triggers spleen contraction [17,18]. Therefore, it is not surprising that voluntary apnea (i.e. hypercapnia and hypoxia) per se is a stimulus for evoking spleen contraction. While the so-called diving reflex is augmented by face immersion, spleen contraction can be seen with and without face immersion [19]. Some studies found increased hemoglobin and hematocrit values after several apneas [4,16,20]. Several authors have speculated that the rapid spleen contraction, which can be seen after even a single apnea, contributes to prolongation of successive repeated apnea attempts [21]. In contrast, we found hemoglobin to be not increased after a maximal apnea. Hemoglobin, hematocrit, MCV, RDW and erythrocytes even dropped significantly 30 min post apnea. Due to the relatively small volume of the spleen, some authors have speculated that the effect of spleen contraction in humans is limited [22].

An increase of reticulocytes might reflect augmented erythropoiesis. This is in line with reported reticulocytes after two weeks of daily apnea training [23]. Although apnea time was 329 ± 103 seconds and SpO_2_ dropped to 79% ± 12%, our data show that in the examined divers, one maximal apnea was not sufficient to trigger erythropoiesis. Physicians should be aware that spleen contraction may play a minor role in adaption, that it requires time and appropriate training or might even be missing [23].

Regarding white blood cells, significant changes were found for neutrophil granulocytes, lymphocytes and monocytes. Some authors have hypothesized that an increase in white blood cells is caused by spleen contraction [20]. Eftedal et al. found changes in gene activity implicating a temporary change of lymphocytes such as cytotoxic t-cells [24]. At our time points, an increase in leucocytes, including neutrophil granulocytes, lymphocytes and monocytes, was detected immediately post apnea followed by a decrease 30 min post apnea and an increase 4 h later. Oxidative stress induced by hypoxia results in neutrophil antioxidant defenses. Sureda et al. showed that repetitive chronic episodes of apnea diving induced adaption mechanisms in neutrophil granulocytes [25]. Metabolic responses of trained breath-hold divers resulted in reduced post-apnea oxidative stress and blood acidosis levels [26]. Lymphocyte antioxidant enzyme activities seems to be induced by exhaustive exercise [27]. In repetitive apneas in trained athletes, similar dynamics of leucocyte subpopulations were reported [20]. Our findings are in line with these observations and in non-elite divers, even a single dry apnea resulted in significant changes of white blood cells involved in antioxidant mechanisms.

Thrombocytes, as key cells of blood clotting, showed no activation of blood coagulation in healthy individuals after exposure to acute hypoxia [28]. Although a significant reduction occurred 30 min after apnea, 4 h values showed no changes compared to baseline values. Baković et al. reported no changes in thrombocytes concentrations in trained persons performing repetitive apneas [20]. Nevertheless, diving response leads to a massive increase of catecholamines, peripheral vasoconstriction and elevated blood pressure >200 mmHg [5,8,10]. The drop of thrombocytes might be caused by consumption due to small micro bleedings.

In conclusion, our study provides new insights for non-elite apnea divers. We could show that postulated adaptive mechanisms regarding cell counts might be not as pronounced as assumed. Considering that apnea diving might substantially burden the cardiovascular system [8], this study underlines the limited adaption performance of humans in case of extended apnea.

### Limitations of the study

To evaluate physiological limits of apneic performance, inclusion criteria were very strict. This resulted in a small absolute sample size. Therefore, some effects could be either hidden or pronounced. Nevertheless, this limitation is also present in comparable studies [23,24].

In this study, apnea was performed under dry conditions. Therefore, immersion effects (i.e. shift of blood volume from extremities to the trunk), drainage of fluids from cells into the vascular lumen, and differences in leukocyte counts as found in divers performing apnea in water might have occurred. Future studies should confirm our results and add further time points for evaluation once values return to baseline. Further repeated apnea might help to better understand additive effects without sufficient washout.

## Supporting information

S1 File Raw data. Supporting information file shows raw data of all blood count parameters at the four time intervals before, post apnea, 0.5 h and 4 h post apnea.
(PDF)

## References

[1] Lindholm P, Lundgren CEG. The physiology and pathophysiology of human breath-hold diving. J Appl Physiol Bethesda Md 1985. 2009;106:284–292. 10.1152/japplphysiol.90991.2008 18974367

[2] Campbell LB, Gooden BA, Horowitz JD. Cardiovascular responses to partial and total immersion in man. J Physiol. 1969;202:239–50 10.1113/jphysiol.1969.sp008807 5770894 PMC1351477

[3] Elia A, Barlow MJ, Deighton K, Wilson OJ, O’Hara JP. Erythropoietic responses to a series of repeated maximal dynamic and static apnoeas in elite and non-breath-hold divers. Eur J Appl Physiol. 2019;119:2557–2565. 10.1007/s00421-019-04235-1 31563983 PMC6858396

[4] Richardson M, de Bruijn R, Holmberg HC, Bjorklund G, Haughey H, Schagatay E. Increase of hemoglobin concentration after maximal apneas in divers, skiers, and untrained humans. Can J Appl Physiol. 2005;30:276–281 10.1139/h05-120 16129892

[5] Eichhorn L, Dolscheid-Pommerich R, Erdfelder F, et al Sustained apnea induces endothelial activation. Clin Cardiol 2017;40:704–709. 10.1002/clc.22720 28464406 PMC6490346

[6] Theunissen S, Sponsiello N, Rozloznik M, Germonpré P, Guerrero F, Cialoni D, et al Oxidative stress in breath-hold divers after repetitive dives. Diving Hyperb Med. 2013;43:63–6. 23813458

[7] Marlinge M, Coulange M, Fitzpatrick RC, Delacroix R, Gabarre A, Lainé N, et al Physiological stress markers during breath-hold diving and SCUBA diving. Physiol Rep. 2019;7:e14033 10.14814/phy2.14033 30912280 PMC6434169

[8] Eichhorn L, Doerner J, Luetkens JA, et al Cardiovascular magnetic resonance assessment of acute cardiovascular effects of voluntary apnoea in elite divers. J Cardiovasc Magn Reson 2018;20:40 10.1186/s12968-018-0455-x 29909774 PMC6004697

[9] Jiang W, Li Y, Sun J, Li L, Li JW, Zhang C, et al Spleen contributes to restraint stress induced changes in blood leukocytes distribution. Sci Rep. 2017 26;7:6501. 10.1038/s41598-017-06956-9 PMC5529540 28747688

[10] Eichhorn L, Erdfelder F, Kessler F, Dolscheid-Pommerich RC, Zur B, Hoffmann U, et al Influence of Apnea-induced Hypoxia on Catecholamine Release and Cardiovascular Dynamics. Int J Sports Med. 2017;38:85–91. 10.1055/s-0042-107351 27454133

[11] Cialoni D, Sponsiello N, Marabotti C, Marroni A, Pieri M, Maggiorelli F, et al Prevalence of acute respiratory symptoms in breath-hold divers. Undersea Hyperb Med. 2012 Jul-Aug;39(4):837–44. 22908840

[12] Boussuges A, Pinet C, Thomas P, Bergmann E, Sainty JM, Vervloet D. Haemoptysis after breath-hold diving. Eur Respir J. 1999 3;13(3):697–9. 10.1183/09031936.99.13369799 10232449

[13] Qvist J, Hill RD, Schneider RC, Falke KJ, Liggins GC, Guppy M, et al Hemoglobin concentrations and blood gas tensions of free-diving Weddell seals. J Appl Physiol (1985). 1986 10;61(4):1560–9. 3096941 10.1152/jappl.1986.61.4.1560

[14] Persson SG, Ekman L, Lydin G, Tufvesson G. Circulatory effects of splenectomy in the horse. I. Effect on red-cell distribution and variability of haematocrit in the peripheral blood. Zentralbl Veterinarmed A. 1973 8;20(6):441–55. 4202916

[15] Vatner SF, Higgins CB, Millard RW, Franklin D. Role of the spleen in the peripheral vascular response to severe exercise in untethered dogs. Cardiovasc Res. 1974 3;8(2):276–82. 10.1093/cvr/8.2.276 4822798

[16] Schagatay E, Haughey H, Reimers J. Speed of spleen volume changes evoked by serial apneas. Eur J Appl Physiol. 2005 1;93(4):447–52 10.1007/s00421-004-1224-0 15503125

[17] Richardson MX, Lodin A, Reimers J, Schagatay E. Short-term effects of normobaric hypoxia on the human spleen. Eur J Appl Physiol. 2008 9;104(2):395–9. 10.1007/s00421-007-0623-4 18043933

[18] Richardson MX, Engan HK, Lodin-Sundström A, Schagatay E. Effect of hypercapnia on spleen-related haemoglobin increase during apnea. Diving Hyperb Med. 2012 3;42(1):4–9. 22437969

[19] Schagatay E, Andersson JP, Nielsen B. Hematological response and diving response during apnea and apnea with face immersion. Eur J Appl Physiol. 2007 9;101(1):125–32. 10.1007/s00421-007-0483-y 17541787

[20] Baković D, Eterović D, Saratlija-Novaković Z, Palada I, Valic Z, Bilopavlović N, et al Effect of human splenic contraction on variation in circulating blood cell counts. Clin Exp Pharmacol Physiol. 2005 11;32(11):944–51. 10.1111/j.1440-1681.2005.04289.x 16405451

[21] Baković D, Valic Z, Eterović D, Vukovic I, Obad A, Marinović-Terzić I, et al Spleen volume and blood flow response to repeated breath-hold apneas. J Appl Physiol (1985). 2003 10;95(4):1460–6. 12819225 10.1152/japplphysiol.00221.2003

[22] Shephard RJ. Responses of the human spleen to exercise. J Sports Sci. 2016;34(10):929–36. 10.1080/02640414.2015.1078488 26287390

[23] Engan H, Richardson M, Lodin-Sundström A, van Beekvelt M, Schagatay E. Effects of two weeks of daily apnea training on diving response, spleen contraction, and erythropoiesis in novel subjects. Scand J Med Sci Sports. 2013;23:340–8. 10.1111/j.1600-0838.2011.01391.x 23802288

[24] Eftedal I, Flatberg A, Drvis I, Dujic Z. Immune and inflammatory responses to freediving calculated from leukocyte gene expression profiles. Physiol Genomics 2016;48:795–802. 10.1152/physiolgenomics.00048.2016 27614202

[25] Sureda A, Batle JM, Tauler P, Cases N, Aguiló A, Tur JA, et al Neutrophil tolerance to oxidative stress induced by hypoxia/reoxygenation. Free Radic Res. 2004;38:1003–9. 10.1080/10715760400000984 15621719

[26] Joulia F, Steinberg JG, Wolff F, Gavarry O, Jammes Y. Reduced oxidative stress and blood lactic acidosis in trained breath-hold human divers. Respir Physiol Neurobiol. 2002;133:121–30. 10.1016/s1569-9048(02)00133-7 12385737

[27] Sureda A, Batle JM, Tauler P, Ferrer MD, Tur JA, Pons A. Vitamin C supplementation influences the antioxidant response and nitric oxide handling of erythrocytes and lymphocytes to diving apnea. Eur J Clin Nutr. 2006;60:838–46. 10.1038/sj.ejcn.1602388 16482080

[28] Mäntysaari M, Joutsi-Korhonen L, Siimes MA, Siitonen S, Parkkola K, Lemponen M, et al Unaltered blood coagulation and platelet function in healthy subjects exposed to acute hypoxia. Aviat Space Environ Med. 2011;82:699–703. 10.3357/asem.3012.2011 21748908

