# Supplementary material for: Hematologic changes after short term hypoxia in non-elite apnea divers under voluntary dry apnea conditions
Source: PLoS One. 2020 Aug 13;15(8):e0237673. doi: 10.1371/journal.pone.0237673 (PMC7425904; doi:10.1371/journal.pone.0237673)
Supplement: S1 File — Supporting information file shows raw data of all blood count parameters at the four time intervals before, post apnea, 0.5 h and 4 h post apnea. (PDF) [file pone.0237673.s001.pdf]

	Baseline	Post apnea	30min post apnea	4 h post apnea
Basophile %	0.60	0.50	0.50	0.50
	1.30	1.20	1.40	0.90
	0.60	0.50	0.50	0.40
	0.50	0.40	0.60	0.40
	1.00	1.10	0.60	0.40
	0.20	0.20	0.30	0.30
	0.40	0.20	0.20	0.10
	2.00	1.40	1.90	1.40
	0.50	0.50	0.60	0.80
	0.90	0.80	0.90	0.80
Basophile abs	0.03	0.04	0.03	0.05
	0.07	0.10	0.07	0.06
	0.03	0.03	0.02	0.02
	0.03	0.03	0.03	0.02
	0.04	0.05	0.02	0.02
	0.01	0.01	0.01	0.02
	0.02	0.01	0.01	0.01
	0.11	0.12	0.08	0.09
	0.04	0.05	0.04	0.07
	0.07	0.07	0.05	0.06
Eosinophile %	2.20	1.40	0.60	0.70
	6.10	3.60	4.60	2.60
	2.80	1.90	1.90	1.90
	1.80	1.30	1.30	1.50
	4.90	3.40	3.60	1.80
	2.00	1.70	1.80	0.90
	2.60	1.40	1.00	0.80
	7.30	5.00	8.20	5.90
	1.40	1.60	1.70	2.50
	1.40	1.30	1.40	1.50
Eosinophile abs	0.12	0.12	0.04	0.07
	0.32	0.30	0.23	0.17
	0.13	0.12	0.08	0.10
	0.11	0.10	0.06	0.08
	0.19	0.16	0.12	0.09
	0.09	0.11	0.07	0.06
	0.12	0.07	0.06	0.06
	0.40	0.43	0.34	0.37
	0.11	0.15	0.11	0.22
	0.11	0.12	0.08	0.11

Erythrocytes	4.70	4.80	4.70	4.60
	5.00	5.20	5.00	5.00
	5.40	5.10	5.00	4.90
	4.30	4.40	4.20	4.30
	4.10	4.30	4.10	4.10
	5.10	5.40	5.10	5.50
	5.20	5.10	4.90	5.20
	4.60	4.50	4.30	4.50
	4.70	4.40	4.30	4.50
	4.20	4.20	4.10	4.10
RDW	11.10	11.00	11.00	11.10
	12.60	12.60	12.60	12.60
	13.10	13.00	12.90	13.00
	11.90	11.70	11.90	11.90
	12.80	12.70	12.80	12.90
	12.00	12.00	12.00	11.90
	12.10	12.00	12.10	12.20
	12.00	11.90	11.90	12.00
	12.90	12.80	12.70	12.90
	12.00	12.00	11.90	12.10
Hematocrit	41	41	40	40
	43	44	42	42
	43	40	39	38
	40	40	38	39
	38	38	37	37
	43	45	42	46
	45	44	43	45
	40	39	37	39
	39	36	35	37
	37	36	36	37
Hemoglobin	14.60	15.20	14.70	14.70
	15.30	15.50	14.90	15.00
	14.30	13.40	13.20	13.10
	14.30	14.50	13.70	14.00
	12.90	13.60	13.10	13.00
	15.60	16.60	15.40	16.80
	15.70	15.30	15.10	15.80
	14.40	14.20	13.50	14.10
	13.00	12.40	12.20	12.50
	13.00	12.90	12.50	12.70

Immature Granulocytes %	0.20	0.20	0.50	0.30
	0.40	0.40	0.40	0.30
	0.40	0.20	0.50	0.20
	0.30	0.30	0.20	0.20
	0.30	0.20	0.00	0.00
	0.20	0.30	0.00	0.10
	0.20	0.20	0.20	0.10
	0.50	0.20	0.50	0.20
	0.30	0.30	0.30	0.10
	0.30	0.20	0.20	0.10

Immature Granulocytes abs	0.01	0.02	0.03	0.03
	0.02	0.03	0.02	0.02
	0.02	0.01	0.02	0.01
	0.02	0.02	0.01	0.01
	0.01	0.01	0.00	0.00
	0.01	0.02	0.00	0.01
	0.01	0.01	0.01	0.01
	0.03	0.02	0.02	0.01
	0.02	0.03	0.02	0.01
	0.02	0.02	0.01	0.01

Immature Reticulocytes	3.20	4.50	3.90	4.00
	8.30	7.80	6.70	6.90
	5.10	5.10	6.70	3.50
	5.60	6.50	6.70	5.70
	5.10	3.50	3.20	3.10
	6.60	7.00	5.40	5.80
	4.20	3.90	4.00	3.10
	6.90	5.50	7.70	4.60
	3.50	3.30	2.40	0.90
	7.20	8.60	9.20	7.80

Immature Granulocytes %	0.20	0.20	0.50	0.30
	0.40	0.40	0.40	0.30
	0.40	0.20	0.50	0.20
	0.30	0.30	0.20	0.20
	0.30	0.20	0.00	0.00
	0.20	0.30	0.00	0.10
	0.20	0.20	0.20	0.10
	0.50	0.20	0.50	0.20
	0.30	0.30	0.30	0.10
	0.30	0.20	0.20	0.10

Immature Granulocytes abs	0.01	0.02	0.03	0.03
	0.02	0.03	0.02	0.02
	0.02	0.01	0.02	0.01
	0.02	0.02	0.01	0.01
	0.01	0.01	0.00	0.00
	0.01	0.02	0.00	0.01
	0.01	0.01	0.01	0.01
	0.03	0.02	0.02	0.01
	0.02	0.03	0.02	0.01
	0.02	0.02	0.01	0.01
Leucocytes	5.39	8.58	6.64	10.42
	5.24	8.39	4.97	6.60
	4.66	6.34	4.20	5.19
	6.03	7.53	4.64	5.31
	3.89	4.75	3.37	5.11
	4.40	6.63	3.86	6.94
	4.67	4.92	5.79	7.53
	5.47	8.68	4.16	6.23
	7.71	9.64	6.52	8.66
	7.67	9.01	5.80	7.18
Lymphocytes %	43.80	42.80	21.80	25.10
	38.00	48.60	33.00	32.30
	36.90	44.20	34.80	32.20
	41.80	49.00	42.00	46.50
	40.90	38.90	34.10	24.70
	29.50	39.40	25.60	22.50
	39.60	40.00	16.80	17.30
	32.40	47.60	27.60	27.10
	30.50	38.60	34.20	34.30
	48.40	52.30	44.80	52.40
Lymphocytes abs	2.36	3.67	1.45	2.62
	1.99	4.08	1.64	2.13
	1.72	2.80	1.46	1.67
	2.52	3.69	1.95	2.47
	1.59	1.85	1.15	1.26
	1.30	2.61	0.99	1.56
	1.85	1.97	0.97	1.30
	1.77	4.13	1.15	1.69
	2.35	3.72	2.23	2.97

	3.71	4.71	2.60	3.76
MCH	31	32	32	32
	31	30	30	30
	26	26	27	27
	33	33	33	33
	31	32	32	32
	31	31	31	31
	30	30	31	30
	31	31	31	32
	28	28	28	28
	31	31	31	31
MCV	87	85	86	87
	85	85	85	85
	79	78	79	78
	93	91	91	92
	91	89	90	90
	86	84	84	85
	86	86	86	87
	87	86	86	86
	83	81	82	82
	88	87	88	89
MCHC	36	37	37	37
	36	35	35	36
	33	34	34	34
	36	36	36	36
	34	35	35	35
	36	37	36	36
	35	35	36	35
	36	36	36	37
	34	35	35	34
	35	35	35	35
Monocytes %	7.20	8.20	5.90	5.80
	7.30	6.90	6.60	4.80
	6.20	6.60	6.70	5.40
	6.80	6.00	5.40	6.80
	10.50	14.30	11.00	10.80
	7.30	7.80	6.70	5.90
	13.10	12.00	7.60	7.60
	7.90	6.80	7.90	8.70

	4.00	4.50	4.00	5.20
	5.50	5.70	5.20	5.20
Monocytes abs	0.39	0.70	0.39	0.60
	0.38	0.58	0.33	0.32
	0.29	0.42	0.28	0.28
	0.41	0.45	0.25	0.36
	0.41	0.68	0.37	0.55
	0.32	0.52	0.26	0.41
	0.61	0.59	0.44	0.57
	0.43	0.59	0.33	0.54
	0.31	0.43	0.26	0.45
	0.42	0.51	0.30	0.37
MPV	9.40	9.70	9.50	9.70
	10.80	11.10	11.00	11.00
	10.40	10.50	10.60	10.40
	10.20	10.00	10.00	10.10
	10.40	10.50	10.60	10.70
	10.10	10.20	10.60	10.10
	10.20	10.30	10.40	10.60
	11.50	11.20	11.10	11.60
	9.20	8.90	9.00	9.20
	12.10	12.10	12.20	12.00
Neutrophile %	46.20	47.10	71.20	67.90
	47.30	39.70	54.40	59.40
	53.50	46.80	56.10	60.10
	49.10	43.30	50.70	44.80
	42.70	42.30	50.70	62.30
	61.00	50.90	65.60	70.40
	44.30	46.40	74.40	74.20
	50.40	39.20	54.40	56.90
	63.60	54.80	59.50	57.20
	43.80	39.90	47.70	40.10
Neutrophile abs	2.49	4.05	4.73	7.08
	2.48	3.33	2.70	3.92
	2.49	2.97	2.36	3.12
	2.96	3.26	2.35	2.38
	1.66	2.01	1.71	3.19
	2.68	3.38	2.53	4.89
	2.07	2.28	4.31	5.59
	2.76	3.41	2.26	3.54

	4.90	5.29	3.88	4.95
	3.36	3.60	2.77	2.88
RPI	0.90	1.00	0.90	0.80
	1.30	1.20	1.30	1.20
	0.70	0.60	0.60	0.60
	1.40	1.40	1.30	1.30
	0.60	0.70	0.60	0.60
	1.60	1.50	1.30	
	0.90	0.90	0.80	0.80
	1.10	1.20	1.00	1.10
	0.70	0.70	0.40	0.70
	1.90	1.80	1.70	1.70
Reticulocytes %	0.98	1.05	1.04	0.89
	1.39	1.27	1.39	1.24
	0.70	0.68	0.73	0.70
	1.55	1.54	1.49	1.51
	0.70	0.81	0.79	0.69
	1.63	1.49	1.44	1.51
	0.91	0.92	0.83	0.79
	1.28	1.37	1.24	1.28
	0.86	0.88	0.84	0.80
	2.26	2.22	2.15	2.10
Reticulocytes abs	45.60	50.50	48.50	41.20
	69.80	65.40	69.20	62.00
	37.90	34.70	36.30	34.30
	67.10	68.10	62.10	64.20
	29.00	34.90	32.70	28.40
	82.60	80.00	72.70	82.70
	47.70	46.80	40.90	41.20
	58.80	62.20	53.80	57.20
	40.10	38.80	36.40	35.70
	95.80	93.00	87.10	86.30
Ret-Hemoglobin	33.90	34.40	34.10	33.90
	32.20	31.90	32.40	32.00
	28.90	28.30	28.90	28.60
	34.70	35.10	34.80	34.30
	32.70	33.30	33.00	32.50
	33.50	33.40	33.20	33.10
	33.00	32.40	32.50	32.30

	33.40	33.30	33.10	33.60
	30.20	30.80	30.60	30.10
	33.10	33.50	33.30	33.00
Thrombocytes	305	342	304	343
	173	197	178	192
	185	186	168	180
	207	212	194	207
	240	255	250	245
	218	246	206	253
	248	238	215	258
	189	188	166	179
	226	217	201	224
	223	204	188	203
